# Alpha-Amylase-Modified Cassava Starch in the Presence of Calcium Lactate as a Sour Starch Substitute for Gluten-Free Breadmaking

**DOI:** 10.3390/foods15173013

**Published:** 2026-08-27

**Authors:** Vanessa Abad-Quevedo, María Jimena Correa, Fabiola Cornejo, Pedro Maldonado-Alvarado

**Affiliations:** 1 Department of Food Science and Biotechnology, Escuela Politécnica Nacional, Quito 170143, Ecuador; vanessa.abad@epn.edu.ec; 2 Centro de Investigación y Desarrollo en Ciencia y Tecnología de los Alimentos (CIDCA), Facultad de Ciencias Exactas, Universidad Nacional de La Plata, Comisión de Investigaciones Científicas de la Provincia de Buenos Aires (CIC), Consejo Nacional de Investigaciones Científicas y Técnicas (CONICET), 47 y 116, La Plata CP 1900, Argentina; mjcorrea@biol.unlp.edu.ar; 3 Facultad de Ingeniería en Mecánica y Ciencias de la Producción, Escuela Superior Politécnica del Litoral (ESPOL), Campus Gustavo Galindo, Km 30.5 Vía Perimetral, Guayaquil 090902, Ecuador; fcornejo@espol.edu.ec

**Keywords:** *Manihot esculenta* Crantz, pasting, amylose content, starch crystallinity, specific volume, starch digestibility

## Abstract

Sour cassava starch is traditionally produced through spontaneous fermentation and solar drying, processes that induce molecular changes responsible for its high baking expansion but are difficult to standardize. Therefore, this study evaluated a sustainable enzymatic strategy to mimic the functional properties of traditional sour cassava starch through the synergistic action of α-amylase (6 U g^−1^) and calcium lactate (CL) (0, 3, 6, and 9 mg/g starch). Structural, rheological, functional, and nutritional properties were evaluated. The results showed that α-amylase (6 U g^−1^) was consistent with preferential modification of the less ordered regions, resulting in a redistribution of short-range molecular organization. The treatment containing 6 U g^−1^ α-amylase and 6 mg/g CL showed lower apparent amylose content, lower retrogradation tendency, higher gelatinization torque, and greater bread specific volume, while maintaining an estimated glycaemic index of 50.9. These findings demonstrate that this enzymatic modification represents a standardized alternative for producing high-quality gluten-free breads.

## 1. Introduction

Sour cassava starch is a fermented, sun-dried starch used as an ingredient in typical breads and biscuits in some South American countries, such as Brazil, Colombia, and Ecuador [1,2,3]. This ingredient is prepared by a traditional process that involves an aqueous extraction of the starch from the ground cassava roots, followed by spontaneous fermentation for 15–30 days in the presence of water in excess and subsequent drying by exposure to the sun for 12–48 h, reaching different grades of acidity [2,4,5]. This process causes macromolecular and molecular changes in starch since the granule structure and crystallinity are affected, and the degree of polymerization of starch polymers is also modified. These changes confer on sour cassava starch the ability to produce doughs that expand and retain gas [3,5]. Thus, it can be used to produce a gluten-free bread without the addition of leavening agents [2]. Despite these properties, traditionally obtained sour cassava starch lacks standardized characteristics due to the inherent variability of the traditional process, resulting in bakery products with varying technological properties, which makes industrial production difficult. As a result, research has extensively focused on mimicking the supramolecular (granular) and molecular degradations typically caused by spontaneous fermentation and solar drying to standardize baking properties, such as specific bread volume, which remains irregular in traditional sour starch production. With the aim to imitate sour cassava starch properties, several approaches have been proposed such as preparation of chemically oxidized starches (using potassium permanganate, sodium hypochlorite, or hydrogen peroxide), lactic acid-acidified starches (either sun-dried or oven-dried) [4], oxidation treatments such as the use of ozone [4,6] application of starters of fermentation [3] and irradiation with ultraviolet radiation [1], among others. The moderate exposure to radiation, UVB, and UVC causes a partial depolymerization of amylose and amylopectin molecules, facilitating the creation of an expandable structure, but excessive radiation (UVC, 9 h) causes too much degradation of starch integrity, leading to undesirable results. These results show that the expansion capacity of cassava starch is intrinsically linked to structural chemical modifications, specifically the formation of carboxylate groups induced by the oxidative processes [1]. Recently, it has been shown that combining fermentation and solar drying in waxy starch reduces the average molar mass and increases the degree of branching, which directly correlates with higher specific bread volume [5].

While chemical and physical methods have shown potential, enzymatic modification offers a more controlled and “green” alternative for starch degradation due to the lack of residues in the product or in the environment [7]. This is particularly important nowadays for consumers concerned about environmental impact. Alpha-amylase, an endoamylase [8] that cleaves internal α-1,4 glycosidic linkages in both amylose and amylopectin, releases glucose, maltose, and dextrin glycosides. The enzyme activity is influenced by the presence of metal ions, such as calcium and magnesium, which can increase it, and by compounds like polyphenols, which act as inhibitors [9,10]. In particular, calcium ions are essential for the catalytic efficiency of alpha-amylases, since every alpha-amylase has at least one Ca^2+^ binding site that serves a structural role in maintaining enzyme integrity. This structural Ca^2+^ is crucial because it connects different domains, ensuring the stability of the active site and, consequently, the overall alpha-amylase activity. In addition to this structural role, some alpha-amylases feature additional binding pockets that interact with free calcium ions. This secondary interaction not only increases the catalytic rate but can also enhance the enzyme’s thermostability [9].

Therefore, regulating enzyme activity at precise cofactor levels is critical when modifying starch properties for specific technological applications. Building on this, his study was conceived as the second stage of a sequential research strategy to develop a controlled alternative to traditional sour cassava starch for gluten-free breadmaking. In a previous study conducted by our research group [11], native cassava starch, traditional sour cassava starch, and α-amylase treatments ranging from 0 to 9 U g^−1^ starch were systematically compared. That work demonstrated that starch modified with 6 U g^−1^ α-amylase exhibited breadmaking performance comparable to traditional sour cassava starch and favorable structural, rheological, functional, and nutritional properties. Based on these findings, the present study focused exclusively on the previously selected enzymatic treatment and investigated whether calcium lactate supplementation could further optimize starch functionality and bread quality. So, the objective of the present work was to evaluate the synergistic effect of calcium lactate and alpha-amylase as a controlled treatment to modify native cassava starch. This approach aims to mimic the structural degradations of traditional sour starch to produce a standardized substitute suitable for high-quality gluten-free breadmaking.

Consequently, traditional sour cassava starch was not included again in the experimental design, as the objective of this work was not to re-establish equivalence with fermented starch but to optimize the performance of the enzymatically modified system.

## 2. Materials and Methods

### 2.1. Raw Material

The yuca (*Manihot esculenta* Crantz) starch, variety INIAP Portoviejo 651, was donated by the National Institute of Agricultural Research (INIAP) Portoviejo experimental station, Ecuador. This yuca variety is derived from clone CM 1335-4 and has a dry matter content of 35.5%, with a yield of 29 to 40 t/ha [12,13].

All reagents were of analytical grade, and alpha-amylase from porcine pancreas (A3176; Type VI-B, ≥5 units/mg solid) was obtained from Sigma-Aldrich (St. Louis, MO, USA) for starch hydrolysis. According to the manufacturer’s specifications, one unit of α-amylase activity is defined as the amount of enzyme that liberates 1.0 mg of maltose from starch in 3 min at pH 6.9 and 20 °C. Additionally, calcium lactate from Sigma-Aldrich (St. Louis, MO, USA; cat. no. 21185) was used.

### 2.2. Starch Treatment

The experimental procedure was adapted from a previously described protocol [5]. In a previous study conducted by our research group, currently accepted for publication (“Evaluation of the Functional and Nutritional Properties of Alpha-Amylase-Modified Cassava Starch in Breadmaking”), the addition of α-amylase at 6 U g^−1^ starch was identified as the optimal concentration for improving the structural, rheological, functional, and nutritional properties of cassava starch. Therefore, this enzyme concentration was selected for the present study.

Initially, the moisture content of native starch (12%) was adjusted to 80%, a condition previously optimized. Each starch sample was divided into two fractions, corresponding to 25.52% and 74.48% of the total weight, respectively. The first fraction was partially pregelatinized by adding 32.26% boiling water, whereas the second fraction was mixed with 67.74% water at 37 °C and manually homogenized. Both fractions were then combined and thoroughly homogenized. Subsequently, α-amylase (6 U g^−1^ starch) and calcium lactate were added at different concentrations (0, 3, 6, and 9 mg/g starch). The enzymatic reaction was carried out at 37 °C for 20 min.

Samples intended for breadmaking and nutritional analyses were directly dried in an oven (Rebelk RS-40, Barcelona, Spain) at 40 °C for 48 h. In contrast, samples intended for structural, rheological, and gelatinization analyses were inactivated by adding 96% ethanol (1 mL/g modified starch) [14], followed by manual homogenization for 1 min. The samples were subsequently dried under the same conditions. All experiments were performed in triplicate using independent replicates.

### 2.3. FTIR

Attenuated Total Reflectance Fourier Transform Infrared (ATR-FTIR) spectra were recorded in the 4000–400 cm^−1^ range using a Jasco 6800 FTIR spectrometer (JASCO Corporation, Tokyo, Japan). Each spectrum was obtained from 25 scans at a resolution of 4 cm^−1^. Data were analysed using Spectrum software Version 1.0.7. This analysis was performed to evaluate potential changes in starch crystallinity and structural order. Specifically, the bands at 1041 cm^−1^ and 994 cm^−1^ (associated with ordered starch) and the band at 1014.5 cm^−1^ (associated with amorphous starch) were examined [3,15].

### 2.4. Measurement of Apparent Amylose Content by Differential Scanning Calorimetry

The amylose content was measured using a differential scanning calorimeter (DSC 204 F1 Phoenix; Netzsch, Selb, Germany) (DSC-LPC). Approximately 5 mg of sample and 50 μL of L-α-lysophosphatidylcholine (Sigma Aldrich, St. Louis, MO, USA) (2% *w*/*w*) were put in an aluminium pan that was hermetically sealed, and an empty pan was used as a reference. Samples were heated from 25 °C to 160 °C at 10 °C/min, maintained at 160 °C for 2 min, and then cooled to 60 °C at 10 °C/min [2]. From the transition corresponding to the amylose-lipid complex, the apparent amylose content was determined during the cooling step [15]. The apparent amylose content was calculated as the ratio between the enthalpy of the sample and the enthalpy of pure amylose. This procedure is based on the formation of complexes between amylose and an added phospholipid (L-a-lysophosphatidylcholine) during cooling.

### 2.5. Determination Pasting Properties

The effect of calcium lactate on starch pasting behavior was evaluated using a Rapid Visco Analyzer (RVA 4500; Perten Instruments, Springfield, IL, USA). Briefly, 3.5 g of samples (on a 14% moisture basis) were added to 25 mL of water and placed into the equipment. Samples were initially stirred at 960 rpm for 10 s and then submitted to a controlled heating profile: heating at 50 °C for 1 min, then heating from 50 °C to 90 °C at 10.8 °C/min, maintained at 90 °C for 4 min, and then cooling from 90 to 50 °C at 10.5 °C/min. Throughout the heating–cooling profile, samples were continuously stirred at 160 rpm. Viscosity changes during the assay were registered, and the following parameters were derived from the curves: pasting temperature (T_p_), peak time (P_t_), peak viscosity (η_p_), minimum viscosity or trough (η_min_), breakdown or instability (η_p_ − η_min_), final viscosity (η_f_), setback from trough (η_f_ − η_min_), and setback from peak (difference between final and peak viscosity).

### 2.6. DeterminationThermo-Rheological Properties

The thermo-rheological properties of the samples were studied using a Mixolab 2 (Chopin Technologies, Villeneuve-la-Garenne, France) following the ‘Chopin+’ protocol, with a hydration level of 80% (previously optimized). The thermo-rheological properties of these samples were evaluated, and parameters related to starch gelatinization (C3), gel stability (C4), and starch retrogradation (total setback, C5-C4) [15] were determined from the curves. Since yuca starch lacks gluten proteins, the parameters C1 and C2 were not evaluated.

### 2.7. Bread Preparation 

The procedure was adapted from a previously reported protocol [2]. The proportions of pregelatinized (25.52%) and non-pregelatinized (74.48%) starch were adopted from this protocol and maintained throughout the study. First, the moisture content of the native starch (NS) was adjusted to 80% (previously optimized value), starting from an initial moisture content of 12%. A total of 25.52% of the native starch was pregelatinized by adding 32.26% of boiling water. Subsequently, the remaining 74.48% of starch was incorporated and mixed with 67.74% water at 37 °C, then manually homogenized until a uniform matrix was obtained. An enzymatic solution equivalent to 6 U of α-amylase (Sigma-Aldrich, A3176, St. Louis, MO, USA) was then added to the suspension, together with different concentrations of calcium lactate (3, 6, and 9 mg/g; Sigma-Aldrich, 21185, Steinheim, Germany), and the mixture was allowed to rest for 20 min at room temperature, a period previously optimized. Dough portions weighing 10 g were hand-kneaded, placed on oven trays, and baked for 11 min at 260 °C in a convection oven (Unox Stefania XFT113, Cadoneghe, Italy). The bread pieces were allowed to cool and stored in bags at room temperature until the following day.

### 2.8. Specific Volume

It was measured 24 h after baking. Firstly, each loaf of bread was weighed (Radwag WTC 2000 scale, Radom, Poland), and then its volume was measured by displacement with birdseed. The specific volume was calculated as the volume divided by the weight of each loaf (cm^3^/g) according to the AACC method 10-05.01 [16]. Three replicates per batch were performed.

### 2.9. Bread Nutritional Properties

Cassava bread samples were ground in a mill (Hamilton Beach, Glen Allen, VA, USA) and passed through a 100 µm mesh sieve to evaluate total starch content and in vitro starch digestibility.

### 2.10. Total Starch Content

It was measured according to AACC Method 76-13.01 [17], using the total starch assay kit of Megazyme (K-TSTA) (Megazyme International, Wicklow, Ireland). Briefly, starch was hydrolysed by a thermostable α-amylase and then by amyloglucosidase, releasing glucose. To measure the released glucose, the glucose oxidase–peroxidase (GOPOD) assay was used. In this assay, D-glucose is oxidized by glucose oxidase to D-gluconate, releasing an equimolar amount of H_2_O_2_, which is quantified by a colorimetric reaction in the presence of peroxidase, which produces a quinone-imine dye. The absorbance of the colored product of this reaction was measured at 510 nm using a microplate reader (BioTek Instruments, Winooski, VT, USA).

### 2.11. In Vitro Starch Digestibility

All samples were processed using the digestible and resistant starch assay kit (K-DSTRS) from Megazyme International (Wicklow, Ireland). This procedure allows the measurement of rapidly digestible starch (RDS), slowly digestible starch (SDS), total digestible starch (TDS), and resistant starch (RS). Briefly, all samples were treated with a mixture of pancreatic α-amylase and amyloglucosidase in maleate buffer (pH 6.0) under continuous stirring at 37 °C for 4 h. The evolution of starch hydrolysis was followed by taking aliquots after 20 min of reaction to measure RDS, after 120 min to measure SDS (as the difference between the starch value at 120 min and the starch value at 20 min), and after 240 min to measure TDS and RS. The aliquots taken for RDS, SDS, and TDS measurements were immediately added to 20 mL of 50 mM acetic acid to stop the enzymatic hydrolysis reaction.

These aliquots were incubated with amyloglucosidase (100 U/mL) (to hydrolyze remaining traces of maltose to glucose), and the released glucose was quantified by the glucose oxidase–peroxidase (GOPOD) assay. To measure the RS, the aliquot was added to an equal volume of ethanol 95%, the mixture was centrifuged, and the remaining pellet was washed with an aqueous solution of ethanol with the aim of removing free glucose. The washed pellet was then suspended in NaOH 1.7 M in an ice/water bath under continuous stirring for 20 min. The solution was neutralized, and the starch hydrolyzed to glucose with amyloglucosidase (3300 U/mL). The released glucose was measured with the GOPOD assay.

The curves of glucose released as a function of time of hydrolysis were fitted according to Equation (1) [18].

(1) $$C=C_{\infty }\;(1-e^{-kt})$$

where *C* is the amount of glucose released at a given time, *t*; $C_{\infty }$ is the equilibrium value (*C* when *t* → ∝); and *k* is the kinetic constant of the starch hydrolysis. To convert glucose into starch, a factor of 0.9 was used. For comparison purposes, the starch hydrolysis of wheat bread was also evaluated under the same conditions as cassava bread. Thus, the hydrolysis index was calculated as the ratio between the area under the curve of cassava bread samples and the area under the curve of wheat bread. On the other hand, the estimated glycaemic index (eGI) was calculated using Equation (2) [19,20].

(2) eGI = 8.198 + 0.862 HI

### 2.12. Statistical Analysis

Statistically significant differences between the mean values of three repetitions were determined using analysis of variance (ANOVA), followed by Fisher’s test with a 95% confidence level. The homogeneity of variances was verified using Levene’s test prior to performing one-way ANOVA. All statistical analyses were performed using Statgraphics Centurion XIX software 19.1.03.

## 3. Results and Discussion

### 3.1. Starch Crystallinity and Apparent Amylose Content

Apparent amylose content and short-range molecular organization are two principal structural attributes that govern starch functionality, as they significantly influence gelatinization, retrogradation, and digestibility. In this study, these attributes were evaluated using complementary analytical techniques. The DSC-LPC method estimates apparent amylose content by measuring the enthalpy of formation of the amylose–lysophosphatidylcholine (LPC) inclusion complex. In contrast, ATR-FTIR spectroscopy provides information on the short-range molecular organization of starch by analyzing absorbance ratios within the 1200–800 cm^−1^ spectral region. These techniques probe distinct hierarchical levels of starch structure and should be considered complementary rather than equivalent measurements of the same structural attribute [21,22].

Enzymatic treatment substantially altered the apparent amylose content as determined by DSC. The 6a+0CL and 6a+3CL treatments exhibited the highest apparent amylose values, while 6a+6CL showed the lowest. Based on the principle of the DSC-LPC method, partial starch hydrolysis likely increased the proportion of linear glucan chains capable of forming inclusion complexes with LPC during cooling, thereby elevating the apparent amylose estimated by this method. In contrast, the lower apparent amylose observed in the 6a+6CL treatment, as shown in Table 1, may result from more extensive hydrolysis of these linear chains, reducing the proportion of molecules with sufficient degree of polymerization to form stable LPC complexes. Thus, the values obtained by DSC should be interpreted as estimates of the fraction of linear glucan chains capable of complexing with LPC, rather than as absolute measurements of amylose content [15].

ATR-FTIR analysis revealed progressive decreases in absorbance intensities at 1041, 1014, and 994 cm^−1^ as calcium lactate concentration increased. While the 1041/1014 ratio remained essentially unchanged among treatments, the 1014/994 ratio decreased significantly, reaching its lowest value in the 6a+6CL treatment. These absorbance ratios are widely recognized as indicators of short-range molecular order and should not be interpreted as direct measurements of absolute starch crystallinity, which is more appropriately assessed by techniques such as X-ray diffraction [21,22].

The reduction in the 1014/994 ratio observed for the 6a+6CL treatment indicates alterations in short-range molecular organization resulting from enzymatic hydrolysis. Less ordered domains of starch granules are generally more accessible to enzymatic attack than the highly organized double-helical structures of amylopectin; therefore, hydrolysis is expected to occur preferentially within these regions during the initial stages of the reaction [23,24,25]. Accordingly, the lower 1014/994 ratio may reflect a redistribution of the relative spectral contributions of less- and more-ordered domains, rather than an absolute change in starch crystallinity [26].

Although the DSC and ATR-FTIR results may initially appear inconsistent, these techniques assess distinct structural characteristics of starch. The DSC-LPC method reflects the availability of linear glucan chains capable of forming inclusion complexes with LPC, whereas ATR-FTIR monitors changes in the short-range molecular organization of the starch matrix. Therefore, the lower apparent amylose content observed in the 6a+6CL treatment does not contradict the decrease in the 1014/994 ratio. Rather, both findings are consistent with more extensive modification of the less ordered regions of the starch granule and a redistribution of short-range molecular organization.

This interpretation is further supported by Pearson correlation analysis, which revealed significant associations between apparent amylose content and the absorbance intensities at 1014 and 1041 cm^−1^, as well as the 1014/994 ratio. Collectively, these results indicate that α-amylase and calcium lactate jointly influenced both the population of linear glucan chains detected by DSC and the short-range molecular organization assessed by ATR-FTIR. Thus, the two techniques provide complementary insights into starch structural modification and help explain, at different structural scales, the rheological, functional, and breadmaking properties observed in subsequent analyses.

### 3.2. Pasting Properties

**Figure 1:**
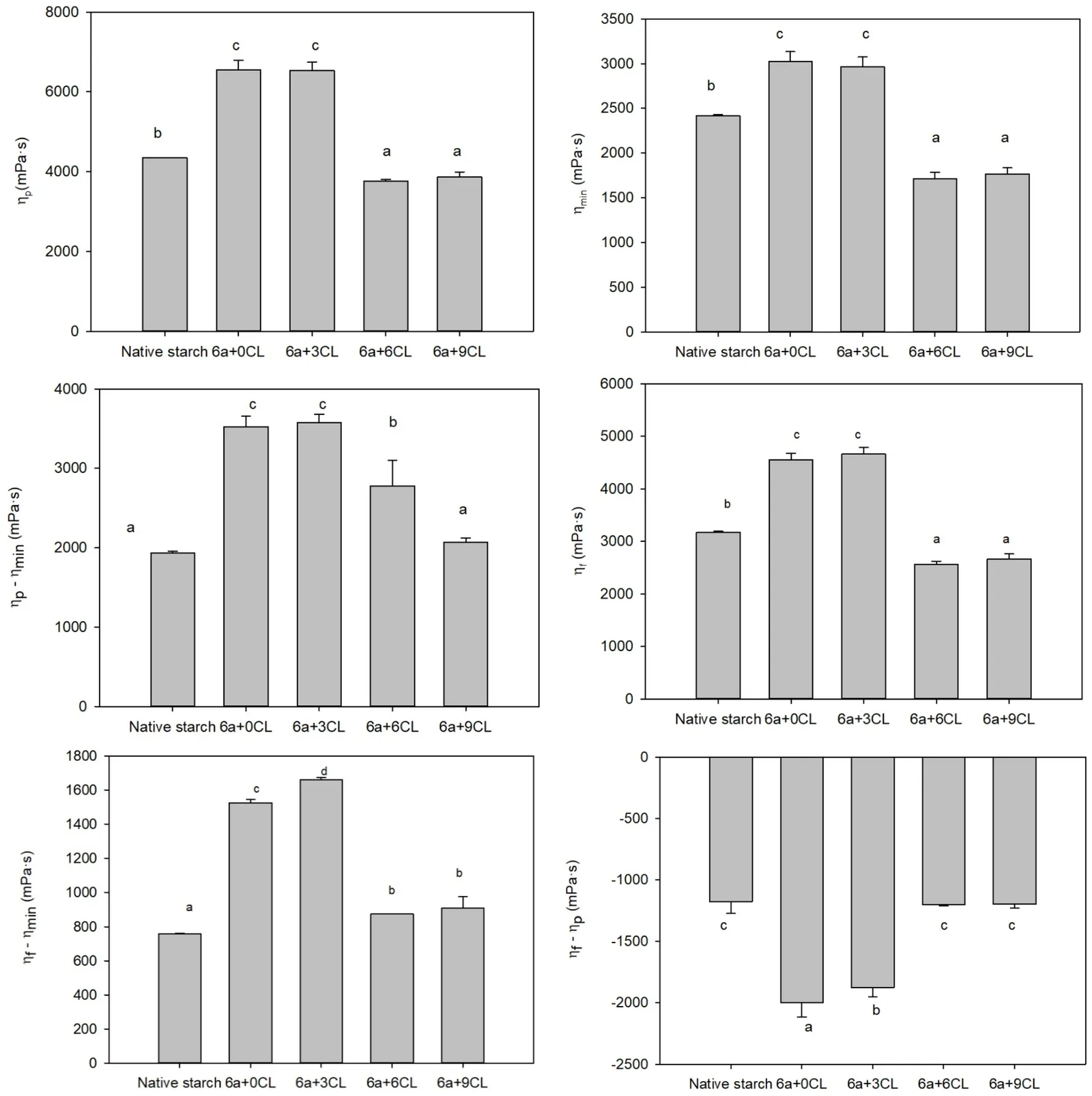
Parameters derived from RVA curves. Values are expressed as mean ± standard deviation (n = 3 independent biological replicates). Different lowercase letters within the same column indicate significant differences according to Fisher’s least significant difference (LSD) test (*p* < 0.05). 6a+0CL: sample with 6 units of amylase and without calcium lactate. 6a+3CL, 6a+6CL, and 6a+9CL denote starch treated with 6 U g^−1^ α-amylase supplemented with 3, 6, and 9 mg g^−1^ calcium lactate, respectively.

Figure 1 shows the parameters obtained from the RVA viscosity curves of the samples. The rapid viscoamylograph is a useful device that allows evaluation of starch behavior under continuous stress (mixing) in the presence of excess water and during a cycle of heating and cooling. Thus, it allows evaluation of starch behavior in a model system when it is cooked in excess water and then subjected to a cooling stage. The temperature at which viscosity starts to increase is called the pasting temperature, which ranged from 70.1 to 70.5 °C for all samples. This increase in viscosity is related to the swelling of starch granules and the complete gelatinization of starch. Thus, the pasting temperature indicates the minimum temperature necessary to cook a flour [27]. After that, at the end of the heating stage, the maximum viscosity is obtained. The peak viscosity (η_p_) parameter obtained from rheological viscosity analysis helps evaluate the gelatinization capacity of starches and is related to the sample’s water-holding capacity. Native cassava starch exhibited ηp midway between the viscosity values of samples treated with alpha-amylase. The sample without calcium lactate and with 3 mg of CL per gram of starch showed the highest values, with no significant differences among them, indicating that this level of CL did not affect amylase activity.

These samples showed higher amorphous content by FTIR (a band at 1014 cm^−1^ and the 1014/994 ratio), which could favor hydration and swelling, resulting in higher viscosity. When higher values of CL were added (6 or 9 mg/g starch), peak viscosity values were lower than those of native starch. This behavior is likely due to increased α-amylase activity facilitated by calcium. Higher activity could cause greater starch degradation, leading to a lower maximum starch viscosity. The higher η_p_ values observed when CaL was added at 0 and 3 mg calcium lactate/g starch indicated that amylase activity modified the starch, leading to a higher water absorption capacity. Since at 6–9 mg/g there were no observed differences in peak viscosity among samples, it could show that at high concentrations, calcium inhibits granule expansion due to ionic competition between cations and hydroxyl groups of starch, which limits its hydration. Thus, the maximum viscosity peak shows a clear downward trend as calcium increases, indicating a threshold dose required to maintain functional gelatinization without compromising matrix integrity.

The minimum viscosity, or trough, is related to the paste’s stability under prolonged heating and stirring. Samples followed the same behavior as observed in the peak viscosity. Samples without CL (6a+0CL) and with 3 mg CL/gr (6a+3CL) presented the highest viscosity, followed by native starch and then by samples with higher levels of calcium lactate. In the same sense, the breakdown, calculated as the difference between the peak and minimum viscosities, is used as a measure of the starch’s susceptibility to disruption under shear stress and heating. A high breakdown value reflects a more brittle structure, while a lower value indicates greater stability. The breakdown was higher for (6a+0CL) and (6a+3CL), and no differences were observed between native starch and the sample with 9 mg of CL/gr of starch. In the case of (6a+6CL) an intermediate value was found. These results could be interpreted as an improvement in thermal resistance when higher levels of CL were used because the drop in viscosity is lower in these samples. However, given the low peak and minimum viscosities, this value could reflect a more limited pasting than a truly better-conserved structure. Thus, the most balanced breakdown was 6a+6CL, reflecting a functional balance between gelatinization and thermal resistance.

The final viscosity, which reflects the formation of a structural network or gel during the cooling stage, followed a trend similar to that of peak viscosity. The highest values were observed in samples 6a+0CL and 6a+3CL, while native starch showed intermediate values, and treatments with 6 and 9 mg CL/g yielded the lowest. These results indicate that samples with higher amylose content developed a more robust final viscosity. Specifically, the treatment without calcium lactate (6a+0CL) achieved the highest amylose content (16.51%), consistent with its high final viscosity and setback, indicating a greater propensity for retrogradation.

The addition of 3 mg/g of CL (6a+3CL) caused a slight reduction in amylose (15.81%); however, this decrease was insufficient to modify thermal stability or significantly reduce retrogradation, as evidenced by the high breakdown and setback values. In contrast, the 6a+6CL sample, which had the lowest amylose value (8.61%), exhibited a substantial reduction in these parameters. This indicates a lower tendency to retrograde and greater functional stability, both of which are highly desirable traits for products requiring a mild texture and prolonged shelf life.

These rheological shifts are supported by a Pearson correlation analysis, which revealed that amylose content significantly influences pasting behavior (*p* < 0.05). Strong positive correlations were found between amylose content and peak viscosity (r = 0.8716), minimum viscosity (r = 0.7331), breakdown (r = 0.7742), final viscosity (r = 0.8469), and setback from minimum (r = 0.9199). Conversely, a strong negative correlation was found with setback from peak (r = −0.9050).

The minimum setback values observed in the 6a+6CL sample—and similarly in 6a+9CL—are directly related to the lower amylose content, which limits gel formation during cooling. Ultimately, the variations in pasting properties among samples can be attributed to the degree of hydrolysis reached, which in turn dictates amylose content, the extent of amylose leaching, and changes in granule rigidity and crystallinity. Because the 6a+9CL sample showed no significant improvement over 6a+6CL, it is suggested that calcium lactate concentrations exceeding 6 mg/g do not provide additional functional benefits to the starch matrix.

### 3.3. Thermo-Rheological Properties

The thermo-rheological analysis conducted with the Mixolab provided further insight into how the synergistic action of alpha-amylase and calcium lactate affects dough consistency during heating and cooling cycles under controlled mixing conditions. The initial part of the Mixolab curve, which pertains to protein behavior during mixing, was excluded from evaluation. Instead, the analysis focused on the evolution of torque as starch underwent modification throughout the temperature protocol. Table 2 shows the results of the parameters derived from Mixolab.

The C3 parameter, which represents the maximum viscosity attained during heating, reflects the capacity of starch to gelatinize and form a viscous structure in the presence of water and heat [28]. Treatment of native starch with 6U enzyme/g starch (6a+0CL) resulted in an 8.4% increase in the C3 parameter, indicating a slight enhancement in water absorption capacity due to hydrolysis. Starches modified with amylase and increasing concentrations of CL (3–6 mg/g) showed a significant increase in this parameter with increasing CL concentration. In particular, the sample with 6 mg of CL/g showed a 30.4% increase relative to native starch. This suggests that fragmented amylose and amylopectin chains have a greater availability of hydroxyl groups, thereby enhancing water absorption [25] and reinforcing the starch gel matrix. These results show that when calcium ions are added in small quantities, starch leads to a stronger matrix. However, at 9 mg/g calcium lactate, the C3 parameter declined, suggesting that higher calcium concentrations may restrict starch swelling or disrupt the starch–water–enzyme interface, causing the dough to resemble native starch and diminishing the benefits of enzyme modification. Previous studies have shown that calcium lactate can delay corn starch swelling, thereby reducing the C3 parameter, and that the impact of salts on starch gelatinization varies with the salt’s chemical nature and concentration [29]. The effect of ions depends on whether they are water structure makers or brokers, as this determines whether there is more or less free water. Furthermore, ions interact with starch via electrostatic forces [30,31]. This interaction can strengthen the starch structure, delaying gelatinization or weakening it. Due to the electronegativity of starch, its interactions with ions are governed by charge density: anions stabilize starch granules by repelling the electronegative -OH groups, whereas cations destabilize the granules by attracting these groups. In both cases, the intensity of the stabilization or destabilization is directly proportional to the specific charge density of the ions involved [30].

The C4 parameter, defined as the minimum viscosity observed during heating at 95 °C and indicative of heat paste stability, showed a slight improvement across all samples except 6a+9CL. Analysis of the C3–C4 difference indicated that samples with 3 and 6 mg of CL per gram of starch exhibited lower hot paste stability, as evidenced by higher values. The C5 parameter, which measures the tendency for retrogradation during the cooling phase, increased progressively with higher calcium lactate concentrations, although a decrease was observed for 6a+9CL. Finally, the C5–C4 difference, which quantifies the propensity for retrogradation during cooling, was highest in the 6a+6CL treatment. Notably, although this sample showed a lower tendency for retrogradation in the RVA test (a setback from the minimum), the Mixolab results indicate that, under the specific mixing and hydration conditions of the “Chopin+” protocol, the 6a+6CL starch forms a more robust network upon cooling. This finding underscores the critical role of water content in the process.

Although the 6a+6CL treatment exhibited the lowest setback values in the RVA test, suggesting a reduced tendency for starch chain reassociation under excess-water conditions, the Mixolab analysis showed the highest C5-C4 value. This apparent discrepancy reflects the fundamentally different experimental conditions and structural phenomena evaluated by the two techniques. RVA characterizes starch pasting and setback in dilute aqueous suspensions, where viscosity changes during cooling are mainly associated with the reassociation of leached starch chains. In contrast, the Mixolab evaluates thermo-mechanical changes in a dough system under limited hydration and continuous shear, where the C5-C4 parameter reflects the combined effects of starch reorganization, water redistribution, and dough matrix development during cooling rather than starch retrogradation alone. Consequently, the higher C5-C4 value observed for the 6a+6CL treatment should not be interpreted as evidence of greater retrogradation per se, but rather as the formation of a more cohesive thermo-mechanical network under the specific conditions of the Mixolab test [28,32,33].

Calcium lactate dissociates into Ca^2+^ and lactate ions; therefore, the observed effects are likely due to multiple concurrent mechanisms. In addition to stabilizing α-amylase and enhancing its catalytic performance, Ca^2+^ may directly influence starch gelatinization through ion–starch interactions, whereas lactate ions may contribute to changes in the system’s ionic environment [9,29,31]. Because the pH of the reaction medium was not monitored during enzymatic treatment, the relative contributions of pH, ionic strength, and specific ion effects cannot be distinguished in the present study. Therefore, the proposed mechanism should be regarded as a plausible interpretation supported by the available evidence rather than as direct experimental confirmation.

### 3.4. Breadmaking

**Figure 2:**
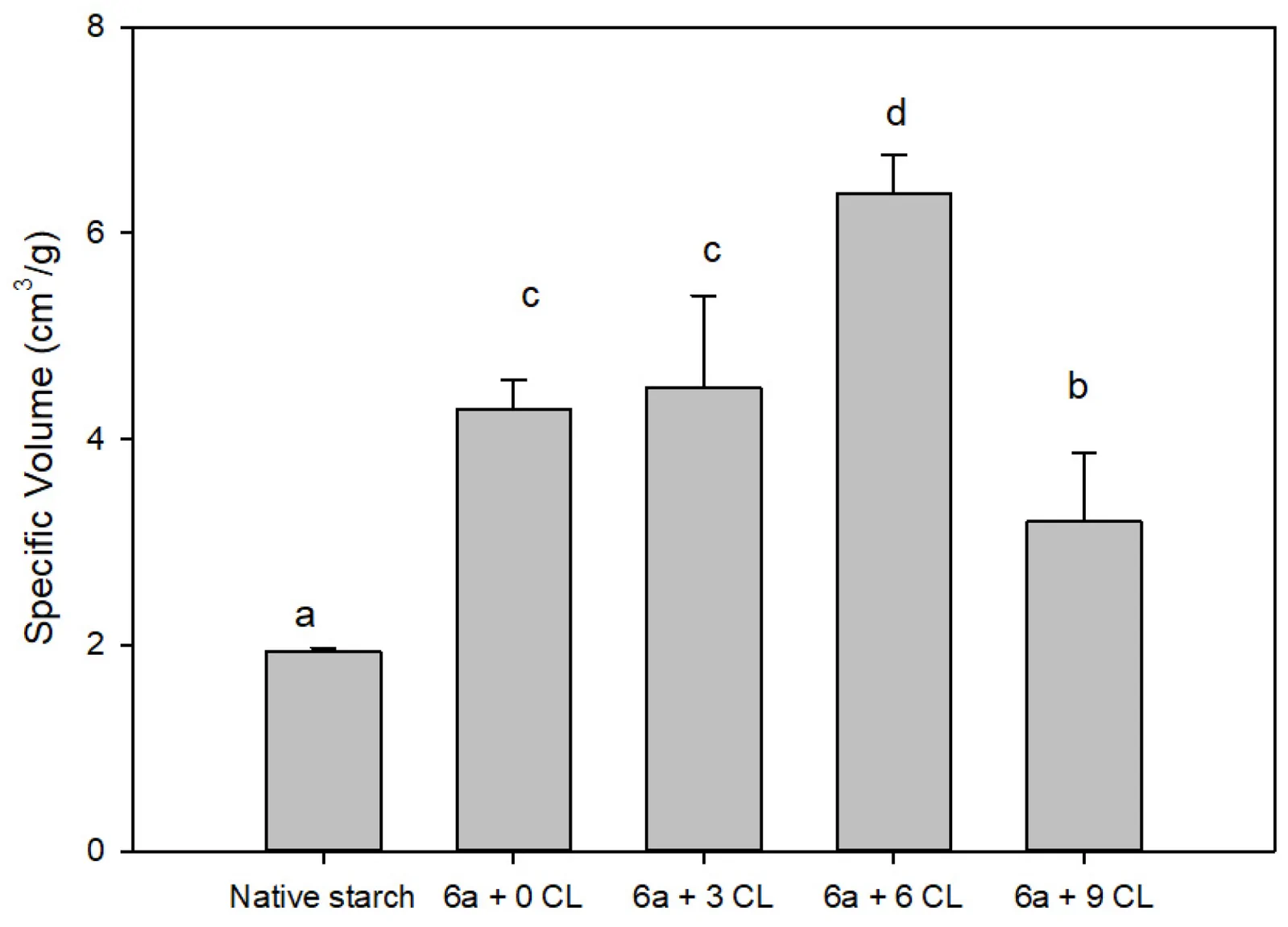
Specific volume of bread. Values are expressed as mean ± standard deviation (n = 3 independent biological replicates). Different lowercase letters within the same column indicate significant differences according to Fisher’s least significant difference (LSD) test (*p* < 0.05). 6a+0CL: sample with 6 units of amylase and without calcium lactate. 6a+3CL, 6a+6CL, and 6a+9CL denote starch treated with 6 U g^−1^ α-amylase supplemented with 3, 6, and 9 mg g^−1^ calcium lactate, respectively.

When evaluating bread quality, the specific volume is a key parameter. Higher volumes are appreciated by consumers because they are associated with softer matrices. The ability of a crumb matrix to retain the gases formed during the fermentation is determinant of the final specific volume. Figure 2 shows that the specific volume increased markedly when cassava starch was treated with alpha-amylase in the presence of calcium lactate. In particular, adding 6 mg of CL yielded the highest specific volume. The lowest value was reached when native starch was used. These results show that partial hydrolysis of starch in sample 6a+6CL had a positive impact on specific volume, whereas higher CL levels (9 mg/g) had a negative impact. This negative effect could be related to CL at this level, as observed in the Mixolab and RVA assays. Particularly, the thermo-rheological behavior observed in the Mixolab was closely reflected in the breadmaking performance of the modified starches. The 6a+6CL treatment, which exhibited the highest gelatinization torque (C3), also produced the highest specific volume among all gluten-free bread samples. This significant increase in C3 for the 6a+6CL sample suggests a superior capacity of starch dough to support the crumb structure during the expansion phase in the oven. In contrast, the 6a+9CL sample showed a marked decrease in specific volume. This aligns with its Mixolab profile, which showed a regression toward native starch values. The excessive concentration of calcium likely restricted granule swelling or induced a different hydrolysis process, resulting in a matrix that could not sustain the gas pressure during baking. These results demonstrated that the synergy between 6 U g^−1^ alpha-amylase and 6 mg/g calcium lactate yields an optimal level of structural degradation. This enzymatic approach is consistent with the bread expansion performance previously reported for α-amylase-modified cassava starch in our previous study [11], while offering the advantage of a standardized and faster process.

### 3.5. Nutritional Properties of Bread

Evaluating starch digestibility is relevant because the rate and extent of glucose release during digestion influence the potential glycaemic behaviour of carbohydrate-rich foods. Based on the kinetics of enzymatic hydrolysis, starch is classified as rapidly digestible starch (RDS, glucose released within 20 min), slowly digestible starch (SDS, glucose released between 20 and 120 min), and resistant starch (RS), which escapes digestion in the small intestine [34]. Originally, RS was defined as the starch remaining undigested after 180 min of hydrolysis [35]. However, following its recognition as dietary fiber by the Codex Alimentarius Commission (CAC), RS is currently defined as the starch fraction that remains undigested after 4 h of hydrolysis, corresponding to the average transit time through the human small intestine [36].

**Figure 3:**
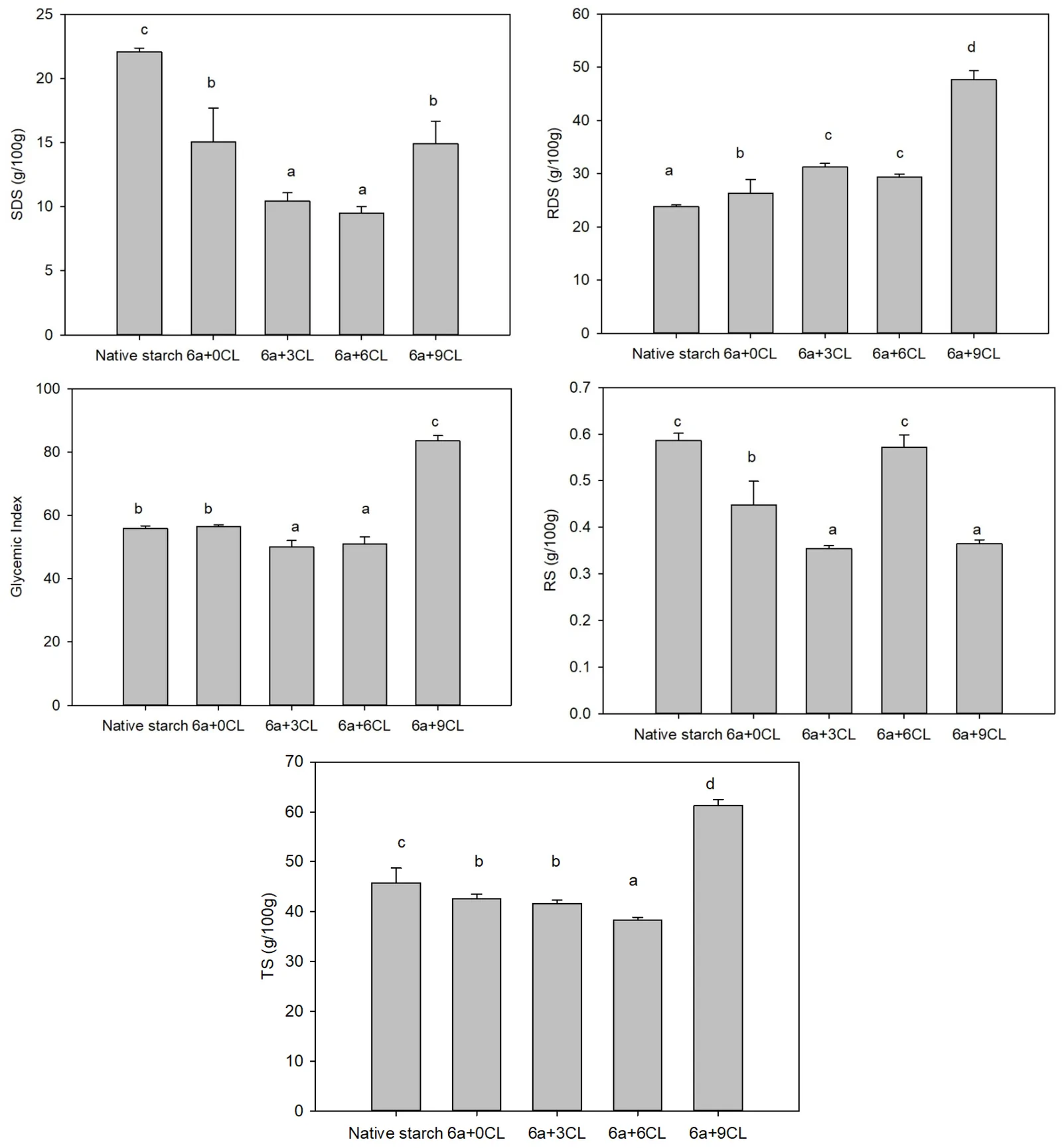
Rapidly digestible starch (RDS), slowly digestible starch (SDS), resistant starch (RS), estimated glycaemic index (eGI) and total starch (TS) of bread samples. Values are expressed as mean ± standard deviation (n = 3 independent biological replicates). Different lowercase letters within the same column indicate significant differences according to Fisher’s least significant difference (LSD) test (*p* < 0.05). 6a+0CL: sample with 6 units of amylase and without calcium lactate. 6a+3CL, 6a+6CL, and 6a+9CL denote starch treated with 6 U g^−1^ α-amylase supplemented with 3, 6, and 9 mg g^−1^ calcium lactate, respectively.

Figure 3 presents the RDS, SDS, RS, and estimated glycaemic index (eGI) of the bread samples. Enzymatic modification of cassava starch in the presence of calcium lactate significantly affected starch digestibility and the estimated glycaemic index. The highest RDS value was observed for the 6a+9CL treatment, suggesting that the more extensive enzymatic modification produced starch structures that were more susceptible to enzymatic digestion. In contrast, native starch exhibited the lowest RDS content, whereas the remaining treatments showed intermediate values.

Slowly digestible starch was also significantly influenced by enzymatic modification. Native starch exhibited the highest SDS content, followed by the 6a+0CL treatment, whereas the 6a+3CL and 6a+6CL treatments showed the lowest values. These results suggest that α-amylase treatment altered the distribution of starch digestibility fractions, decreasing the proportion of starch hydrolysed during the intermediate stage of digestion.

Resistant starch remained below 1 g/100 g in all bread samples, indicating that, despite the statistically significant differences among treatments, the breads remained highly digestible overall. The native starch and 6a+6CL treatments exhibited the highest RS contents, whereas the 6a+3CL and 6a+9CL treatments showed the lowest values. Although the 6a+6CL treatment retained an RS content comparable to that of the native starch, the absolute amount of resistant starch remained very low and is therefore unlikely to substantially influence the overall digestibility of the breads.

The estimated glycaemic index was also significantly affected by calcium lactate supplementation. Breads prepared with the 6a+3CL and 6a+6CL starches exhibited the lowest eGI values, whereas the 6a+9CL treatment showed the highest value (83.56). Because the eGI was derived from an in vitro digestion model, these results should be interpreted as indicative of differences in the potential glycaemic behavior of the breads rather than as direct evidence of the glycaemic response in humans. Moreover, given that the resistant starch content remained below 1 g/100 g across all formulations, the observed reductions in eGI reflect relative differences in the in vitro starch digestion profile rather than clinically relevant reductions in starch digestibility or postprandial glycaemic response.

Finally, the total starch content ranged from 38.26 g/100 g in the 6a+6CL treatment to 61.23 g/100 g in the 6a+9CL treatment. These differences may be associated with the extent of starch hydrolysis during α-amylase treatment and with differences in moisture loss during baking. Greater water loss would increase the proportion of starch on a fresh-weight basis, which may explain the higher total starch content observed in the 6a+9CL bread.

## 4. Conclusions

The results of this study demonstrate that the synergistic action of alpha-amylase and calcium lactate is an effective “green” strategy to mimic the functional properties of traditional sour cassava starch for gluten-free breadmaking. The combined DSC and ATR-FTIR results are consistent with preferential modification of the less ordered regions of the starch granule, resulting in a redistribution of short-range molecular organization. The 6a+6CL treatment (6 U g^−1^ of enzyme and 6 mg/g of CL) was identified as the optimal modification level, providing a critical balance between structural degradation and functional integrity. This sample exhibited the highest gelatinization torque (C3) in the Mixolab, which directly translated into the highest bread specific volume. Furthermore, this treatment maintained a controlled glycaemic profile, with one of the lowest estimated glycaemic indices and resistant starch levels comparable to native starch. In contrast, exceeding this threshold (9 mg/g of CL) led to over-modification, negatively impacting both the dough’s expansion capacity and the bread’s nutritional quality by significantly increasing its eGI. Consequently, the 6a+6CL modification represents a standardized enzymatic alternative for gluten-free breadmaking, offering enhanced technological performance and a more favorable in vitro starch digestibility profile.

## Figures and Tables

**Table 1:** Apparent amylose content and FTIR structural indices.

Sample	Apparent Amylose (%)	1041 cm^−1^	1014 cm^−1^	1041/1014	994 cm^−1^	1014/994
Native Starch	9.32 ± 0.02 ^b^	1.241 ^d^	1.244 ^d^	0.997 ^a^	1.358 ^d^	0.9160 ^c^
6a+0CL	16.51 ± 0.08 ^e^	1.457 ^e^	1.462 ^e^	0.996 ^a^	1.568 ^e^	0.9327 ^e^
6a+3CL	15.81 ± 0.02 ^d^	1.224 ^c^	1.228 ^c^	0.997 ^a^	1.324 ^c^	0.9275 ^d^
6a+6CL	8.61 ± 0.03 ^a^	1.076 ^b^	1.08 ^b^	0.996 ^a^	1.283 ^b^	0.8417 ^a^
6a+9CL	12.53 ± 0.02 ^c^	0.938 ^a^	0.942 ^a^	0.995 ^a^	1.066 ^a^	0.8836 ^b^

Values are expressed as mean ± standard deviation (*n* = 3 independent biological replicates). The coefficient of variation for the FTIR measurements was lower than 0.24% for all samples. Different lowercase letters within the same column indicate significant differences according to Fisher’s least significant difference (LSD) test (*p* < 0.05). 6a+0CL: sample with 6 units of amylase and without calcium lactate. 6a+3CL, 6a+6CL, and 6a+9CL denote starch treated with 6 U g^−1^ α-amylase supplemented with 3, 6, and 9 mg g^−1^ calcium lactate, respectively.

**Table 2:** Mixolab parameters.

Sample	C3 (Nm)	C4 (Nm)	C3-C4 (Nm)	C5 (Nm)	C5-C4 (Nm)
Native Starch	1.54 ± 0.006 ^a^	0.95 ± 0.03 ^a^	0.59 ± 0.03 ^a^	1.89 ± 0.03 ^a^	0.94 ± 0.02 ^a^
6a+0CL	1.67 ± 0.03 ^b^	1.11 ± 0.02 ^b^	0.55 ± 0.02 ^a^	2.19 ± 0.1 ^b^	1.07 ± 0.1 ^a^
6a+3CL	1.85 ± 0.1 ^c^	1.12 ± 0.02 ^b^	0.72 ± 0.1 ^b^	2.24 ± 0.2 ^b^	1.12 ± 0.2 ^a^
6a+6CL	2.01 ± 0.07 ^d^	1.13 ± 0.01 ^b^	0.88 ± 0.07 ^c^	2.62 ± 0.1 ^c^	1.48 ± 0.1 ^b^
6a+9CL	1.57 ± 0.008 ^ab^	0.98 ± 0.05 ^a^	0.58 ± 0.04 ^a^	1.96 ± 0.05 ^a^	0.98 ± 0.006 ^a^

Values are expressed as mean ± standard deviation (n = 3 independent biological replicates). Different lowercase letters within the same column indicate significant differences according to Fisher’s least significant difference (LSD) test (*p* < 0.05). 6a+0CL: sample with 6 units of amylase and without calcium lactate. 6a+3CL, 6a+6CL, and 6a+9CL denote starch treated with 6 U g^−1^ α-amylase supplemented with 3, 6, and 9 mg g^−1^ calcium lactate, respectively.

## Data Availability

The original contributions presented in this study are included in the article. Further inquiries can be directed to the corresponding author.

## Abbreviations

The following abbreviations are used in this manuscript:

CL	Calcium lactate
RVA	Rapid Visco Analyzer
FTIR	Fourier Transform Infrared Spectroscopy
ATR	Attenuated Total Reflectance
DSC	Differential Scanning Calorimetry
LPC	lysophosphatidylcholine
RDS	Rapidly Digestible Starch
SDS	Slowly Digestible Starch
RS	Resistant Starch
TDS	Total Digestible Starch
eGI	Estimated Glycaemic Index
